# New York Code of Ethics

**Published:** 1882-04-20

**Authors:** 


NEW YORK CODE OF ETHICS.—The action of the Medical Society of New York, in authorizing consultations with *all legally qualified* practitioners, will probably be discussed and condemned at the next meeting of the American Medical Association.

